# Dosimetric impact of dental metallic crown on intensity‐modulated radiotherapy and volumetric‐modulated arc therapy for head and neck cancer

**DOI:** 10.1120/jacmp.v17i1.5870

**Published:** 2016-01-08

**Authors:** Takeshi Kamomae, Yoshiyuki Itoh, Kuniyasu Okudaira, Takayoshi Nakaya, Masashi Tomida, Yoshikazu Miyake, Hiroshi Oguchi, Takehiro Shiinoki, Mariko Kawamura, Noriyuki Yamamoto, Shinji Naganawa

**Affiliations:** ^1^ Department of Therapeutic Radiology Nagoya University Graduate School of Medicine Nagoya Japan; ^2^ Department of Radiological Technology Nagoya University Hospital Nagoya Japan; ^3^ Department of Radiological and Medical Laboratory Sciences Nagoya University Graduate School of Medicine Nagoya Japan; ^4^ Department of Therapeutic Radiology Yamaguchi University Graduate School of Medicine Ube Japan; ^5^ Department of Radiology Nagoya University Graduate School of Medicine Nagoya Japan; ^6^ Department of Oral and Maxillofacial Surgery Nagoya University Graduate School of Medicine Nagoya Japan

**Keywords:** dental metallic crown, intensity‐modulated radiotherapy, volumetric‐modulated arc therapy, dose enhancement, head and neck cancer

## Abstract

Metal dental restoration materials cause dose enhancement upstream and dose disturbance downstream of the high‐density inhomogeneous regions in which these materials are used. In this study, we evaluated the impact of a dental metallic crown (DMC) on intensity‐modulated radiotherapy (IMRT) and volumetric‐modulated arc therapy (VMAT) for head and neck cancer. Additionally, the possibility of sparing the oral mucosa from dose enhancement using an individual intraoral mouthpiece was evaluated. An experimental oral phantom was designed to verify the dosimetric impact of a DMC. We evaluated the effect on single beam, parallel opposing beam, arc beam, IMRT, and VMAT treatment plans. To evaluate the utility of a 3‐mm‐thick intraoral mouthpiece, the doses across the mouthpiece were measured. For single beam irradiation, the measured doses at the entrance and exit planes of the DMC were 51% higher and 21% lower than the calculated dose by the treatment planning system, respectively. The maximum dose enhancements were 22% and 46% for parallel opposing beams and the 90° arc rotation beam, respectively. For IMRT and VMAT, the measured doses adjacent to the DMC were 12.2%±6.3% (mean±1.96 SD) and 12.7%±2.5% higher than the calculated doses, respectively. With regard to the performance of the intraoral mouthpiece for the IMRT and VMAT cases, the disagreement between measured and calculated doses at the outermost surface of the mouthpieces were −2.0%, and 2.0%, respectively. Dose enhancements caused by DMC‐mediated radiation scattering occurred during IMRT and VMAT. Because it is difficult to accurately estimate the dose perturbations, careful consideration is necessary when planning head and neck cancer treatments in patients with DMCs. To spare the oral mucosa from dose enhancement, the use of an individual intraoral mouthpiece should be considered.

PACS numbers: 87.55.km, 87.55.N‐, 87.55.Qr

## I. INTRODUCTION

Radiation therapy (RT) has been widely adopted for head and neck cancers. Compared with conventional RT, intensity‐modulated RT (IMRT) has better survival outcomes and less treatment‐related side effects.^1^, ^2^, ^3^, ^4^, ^5^ However, both have radiation‐associated toxicities that can result in poor patient quality of life. For head and neck irradiation, in most cases, the mucosa of the oral cavity is included in the radiation fields, and oral mucositis is an inevitable complication of RT to the head and neck region. Therefore, to complete a treatment on schedule, it is important to minimize oral mucositis.^(6)^

RT of the head and neck region is often administered to patients with nonremovable metal dental restoration materials. There are various types of dental restoration materials, including dental fillings, crowns, bridges, and implants. We have previously shown that a dental metallic crown (DMC) caused dose enhancement upstream of DMC regions because of dose backscatter, and that dose disturbance occurred downstream of DMC regions.^(7)^ Dose enhancement at the incident surface of the DMC was of particular concern. In addition, several studies have evaluated the impact of metal dental restoration materials on simple experimental square‐ or rectangular‐field beams.^8^, ^9^, ^10^, ^11^ For clinically relevant field beams, metal dental restoration materials also have been shown to have considerable dose‐altering effects.^12^, ^13^, ^14^ Shimamoto et al.^(15)^ reported on dose enhancement in the direction of the buccal mucosa due to various dental metal materials for three‐dimensional conformal radiation therapy and IMRT. In another study, Mail et al.^(16)^ showed dose perturbation in both downstream and upstream directions at the surfaces of dental filling materials with volumetric‐modulated arc therapy (VMAT). These unexpected dose perturbations have the potential to cause poor local tumor control and/or serious radiation‐associated toxicities.

Currently, there have been few studies evaluating the impact of metal dental restoration materials on IMRT and VMAT, and these studies mainly involved dental restoration materials measuring about 10 mm in diameter or side‐length. In this study, to imitate the full cast crown, we used an experimental oral phantom in which thin dental materials were embedded, because DMCs in general have a maximum thickness of 1 mm.^(15)^ The aim of the present study was to evaluate the impact of a DMC on IMRT and VMAT for the treatment of head and neck cancer. To explore the possibility of estimating dose perturbations from the doses calculated using treatment planning systems (TPSs), correlations between the calculated doses and the measured dose enhancements adjacent to a DMC were assessed. In addition, we investigated the utility of an individual intraoral mouthpiece to protect the oral mucosa from dose enhancements.

## II. MATERIALS AND METHODS

### A. Experimental oral phantom description

**Figure 1:**
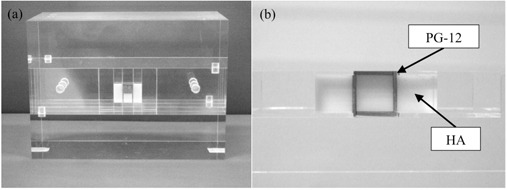
Experimental oral phantom (a) comprising a dental alloy plate (PG‐12) and hydroxyapatite (HA) embedded in polymethyl methacrylate; and (b) the axial view of the phantom.

A phantom was specifically designed to verify the dosimetric impact of a DMC, as shown in Fig. 1. The phantom was a polymethyl methacrylate cube (Mashinax Co., Nagoya, Japan) with a mock metal crown and teeth. The mock metal crown and tooth materials were dental alloy (PG‐12: 12% Au, 20% Pd, 54% Ag, 12% Cu, 1% Zn, and 1% Ru; effective atomic number $[Z_{\text{eff}}]=52.72$, physical density $[\rho ]=11.0\;g\;{\text{cm}}^{-3}$; Cendres+Métaux SA, Biel/Bienne, Switzerland) and hydroxyapatite (HA: 5.891% H, 50.070% C, 3.197% N, 22.916% O, 5.678% P, and 12.247% Ca; $Z_{\text{eff}}=11.66,\;\rho =1.303\;g\;{\text{cm}}^{-3}$; Kyoto Kagaku Co., Kyoto, Japan), respectively. The thickness of the PG‐12 plate was 1 mm. To imitate the full cast crown, five plates of PG‐12 were used. Radiochromic films could be placed in the phantom on the central axis, opposite the metal crown, and on either side of the metal crown. Other phantom details have been described previously.^(7)^

### B. Dose measurements and calculations

All measurements in this study were based on the use of 6 MV photon beams produced using a Clinac iX linear accelerator (Varian Medical Systems, Palo Alto, CA). The delivered irradiation doses were measured using radiochromic films (GafChromic EBT3; Ashland Inc., Covington, KY). To establish a calibration curve of film pixel values versus dose values, 17 films were irradiated with a dose of 0–400 cGy at a depth of 10 cm in a water‐equivalent phantom (Tough Water; Kyoto Kagaku Co., Kyoto, Japan). Films were left for 24 hours before scanning to avoid a postexposure density‐growth effect.^(17)^ Films were scanned using an ES‐10000G flatbed scanner (Seiko Epson Corp., Nagano, Japan) with a resolution of 150 dots per inch in 48‐bit RGB mode. The recommendations of the American Association of Physicists in Medicine (AAPM) Task Group 55 were followed for radiochromic film dosimetry.^(18)^ Digitized data in the red color channel were analyzed using a DD‐System (R‐TECH Inc., Tokyo, Japan).^(19)^ Measured doses were compared to the calculated doses for TPS (Eclipse Ver. 8.9; Varian Medical Systems). The streaking in computed tomography (CT) images near the DMC was corrected according to the Hounsfield unit (HU) value of the polymethyl methacrylate cube.^(20)^ All dose calculations were performed using the anisotropic analytical algorithm (AAA; Varian Medical Systems).

### C. Non‐IMRT field studies

**Figure 2:**
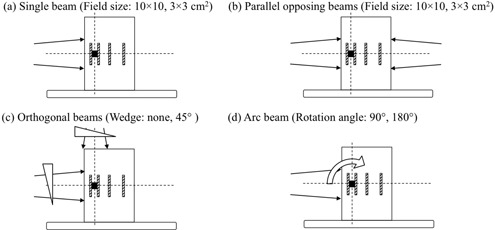
Positional relationships between the radiation beams, isocenter point (intersection of dashed lines), dental metallic crown (black cube), and films (striped diagonal lines).

To evaluate the basic properties underlying the impact of the DMC, the phantom was irradiated using the following settings: (a) a single beam with different field sizes (10×10 and $3\times 3\;{\text{cm}}^{2}$); (b) parallel opposing beams with different field sizes (10×10 and $3\times 3\;{\text{cm}}^{2}$); (c) orthogonal beams with and without a 45° physical wedge; and (d) arc beams at different gantry rotation angles (90° and 180°). The positional relationships between the beams, DMC, and films are shown in Fig. 2.

### D. IMRT and VMAT planning

To assess the effect of a DMC on the IMRT and VMAT treatment fields, test and clinical case plans were evaluated. The evaluations were performed using a nine‐field IMRT and two‐arc VMAT. The two‐arc VMAT was performed using 181°–179° clockwise and 179°–181° counterclockwise rotations.

**Figure 3:**
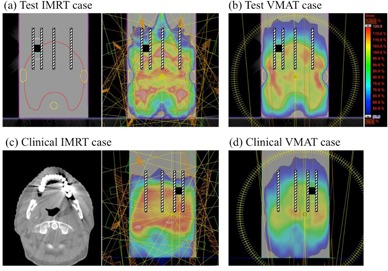
Positional relationships between the TPS calculated dose distribution, dental metallic crown (black cube), and films (striped diagonal lines). The test IMRT (a) and VMAT (b) cases followed the planning concepts in the AAPM TG‐119 report.^(21)^ Examples of clinical IMRT (c) and VMAT (d) cases are shown.

For test cases, the dose prescription and dose goals followed the constraints described in the AAPM Task Group 119 report.^(21)^ Among the clinical cases, three patients who previously underwent IMRT at our institution were selected. The clinical characteristics of the patients are summarized in Table 1. The study was approved by the Institutional Review Board of Nagoya University Hospital, and all patients provided written informed consent. In all cases, VMAT plans were reoptimized to obtain the same dose goals as IMRT plans. The positional relationships between the dose distribution, DMC, and films are shown in Fig. 3. When treatment plans for the patient were applied to the phantom CT dataset, the isocenter positions were decided such that the 95% isodose lines intersected the DMC.

To explore the possibility of estimating dose perturbations from the doses calculated using TPSs, correlations between the calculated doses and the measured dose enhancements adjacent to a DMC were evaluated. First, seven verification plans per patient were created. In the verification plans, the isocenter positions were shifted from the original position by +2.0, +1.0, +0.5, 0.0, −0.5, −1.0, and −2.0 cm in the anterior–posterior (AP) direction; other delivery parameters were not altered. Thus, the calculated doses adjacent to the DMC varied across all verification plans. The verification plans for VMAT were generated in the same manner as those for IMRT. To statistically examine the relationship between the calculated and measured doses, Pearson's correlation coefficients were computed.

**Table 1:** Patient characteristics.

*Patient*	*Sex*	*Age (years)*	*Primary Site*	*Clinical TNM*
1	Male	43	Oropharynx	cT4N2bM0
2	Male	54	Epipharynx	cT2N0M0
3	Female	53	Oropharynx	cT4N2cM0

### E. Validation of an intraoral mouthpiece to protect the oral mucosa

To evaluate the utility of an individual intraoral mouthpiece in protecting the oral mucosa from dose enhancements, a mouthpiece sheet (Erkoflex; Erkodent Erich Kopp GmbH, Germany) was used in conjunction with the phantom. The thickness of each mouthpiece sheet was 1 mm, and three sheets were stacked on the DMC. A thickness of 3 mm was chosen based on our previous study, in which the region in the depth direction with dose enhancement was limited to 3 mm from the surface of the DMC.^(7)^ Radiochromic films were placed between mouthpiece sheets. The phantom mouthpiece setup was irradiated using single beam, parallel opposing beams, IMRT plans, and VMAT plans.

## III. RESULTS

**Figure 4:**
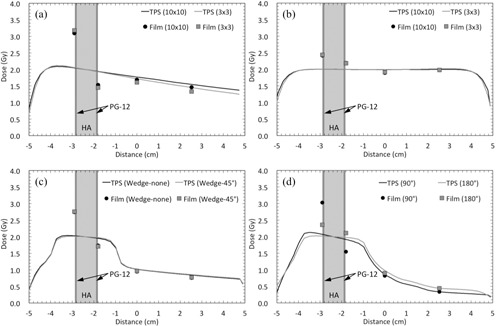
Comparison between the calculated and measured doses for the dental alloy plate (PG‐12) and hydroxyapatite (HA) in non‐IMRT field studies. Profiles for (a) single beams with different field sizes (10×10 and $3\times 3\;{\text{cm}}^{2}$), (b) parallel opposing beams with different field sizes (10×10 and $3\times 3\;{\text{cm}}^{2}$), (c) orthogonal beams with and without the 45° wedge, and (d) arc beams at different gantry rotation angles (90° and 180°).

Figure 4 shows the comparison between the calculated and measured doses for non‐IMRT field studies. For the single beam plan using a $3\times 3\;{\text{cm}}^{2}$ field size, the measured dose at the entrance plane of the DMC was 54% higher than the calculated dose. Conversely, the measured dose at the exit side was 25% lower than the calculated dose. The maximum dose enhancements were 22%, 37%, and 46% for the parallel opposing beams ($3\times 3\;{\text{cm}}^{2}$), orthogonal beams with the 45° wedge, and the arc beams at gantry rotation angle of 90°, respectively.

**Figure 5:**
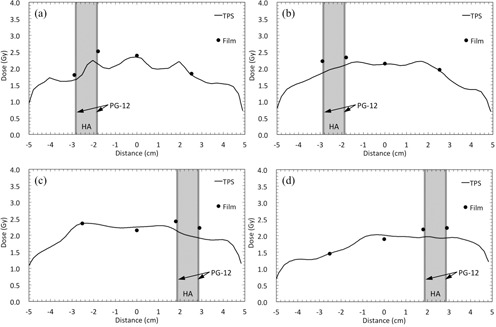
Comparison between the calculated and measured doses across the dental alloy plate (PG‐12) and hydroxyapatite (HA) for IMRT and VMAT. Profiles for (a) test IMRT, (b) test VMAT, (c) clinical IMRT, and (d) clinical VMAT plans.

Figure 5 shows the comparisons between the calculated and measured doses for IMRT and VMAT. The measured doses on either side of the DMC were higher than the calculated doses. The maximum dose enhancements in the test IMRT, test VMAT, clinical IMRT (Patient 1), and clinical VMAT (Patient 1), were 18%, 19%, 14%, and 16%, respectively.

**Figure 6:**
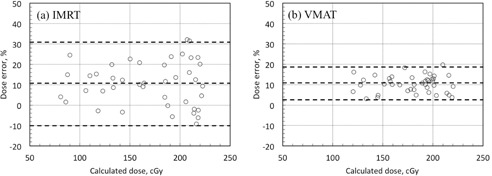
Plots of percentage dose error versus calculated dose at the point of measurement adjacent to the DMC for IMRT (a) and VMAT (b). The top and bottom dashed lines show the 95% confidence level (1.96 SD). The middle dashed line shows the mean dose error.

For all verification plans created by shifting isocenter positions, the measured doses on either side of the DMC were 10.8%±21.0% (mean±1.96 SD) and 10.4%±8.1% higher than the calculated doses for IMRT and VMAT, respectively (Fig. 6). The variation in dose error for VMAT was significantly smaller than that for IMRT (p<0.001).

**Figure 7:**
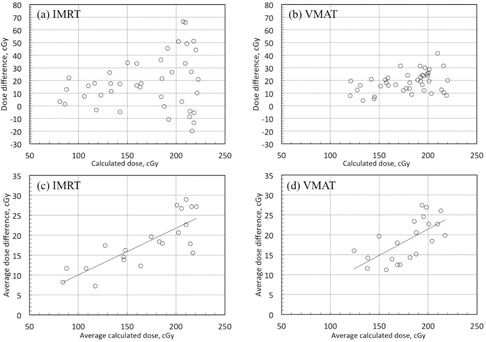
Plot of calculated doses versus measured dose differences ((a): IMRT, (b): VMAT), and average calculated dose on both side of the DMC versus average measured dose differences on both sides of the DMC ((c): IMRT, (d): VMAT).

There were no strong correlations between the dose enhancements (measured doses minus calculated doses) and the calculated doses (IMRT: r=0.20, p=0.20; VMAT: r=0.38, p=0.01; Figs. 7(a) and 7(b), respectively). However, there was a clear correlation between the average calculated dose on both sides of the DMC and the average dose enhancement on both sides of the DMC (IMRT: r=0.81, p<0.001; VMAT: r=0.66, p=0.001; Figs. 7(c) and 7(d), respectively).

**Figure 8:**
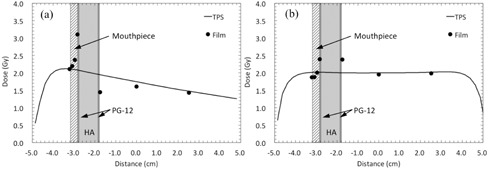
Comparison between the calculated and measured doses across the dental alloy plate (PG‐12), hydroxyapatite (HA), and mouthpiece. The thickness of mouthpiece was 3 mm. Profiles for (a) single beam with a $3\times 3\;{\text{cm}}^{2}$ field size, and (b) parallel opposing beams with a $3\times 3\;{\text{cm}}^{2}$ field size.

**Figure 9:**
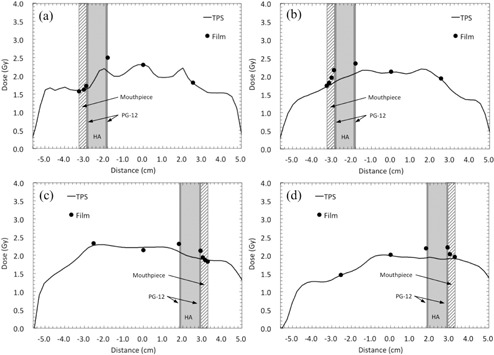
Comparison between the calculated and measured doses across the dental alloy plate (PG‐12), hydroxyapatite (HA), and 3‐mm‐thick mouthpiece. Profiles for (a) test IMRT, (b) test VMAT, (c) clinical IMRT, and (d) clinical VMAT plans.

For the sparing capability of an intraoral mouthpiece, the measured doses at the outermost surface of the mouthpieces were 0.3% and 6.8% lower than the calculated doses for the single and parallel opposing beams, respectively (Figs. 8(a) and 8(b)). For the IMRT and VMAT plans, the disagreement between measured and calculated doses at the outermost surface of the mouthpieces in the test IMRT, test VMAT, clinical IMRT (Patient 1), and clinical VMAT (Patient 1) plans were −1.1%, 1.5%, −2.0%, and 2.0%, respectively, as shown in Fig. 9.

## IV. DISCUSSION

For single beam irradiation, the measured dose at the entrance plane of the DMC was enhanced, and that at the exit plane was reduced. Therefore, dose enhancement and reduction with parallel opposing beams were lower than those with single beam irradiation. The basic characteristics for single and opposing beams were consistent with previously published data.^(7)^ The results in present study clearly showed that the use of multiple beams and wide gantry rotation angles could mitigate the dosimetric impact of a DMC. For IMRT and VMAT, dose enhancement and/or reduction occurred “randomly” because of the complex combination of the beams penetrating the DMC, although the dose enhancements and reductions for VMAT were smaller than those for IMRT. In the case of VMAT, the interaction between dose enhancement and reduction was uniform. The difference in the uniformity of the beam incident angle (IMRT: fixed gantry angle, VMAT: full gantry rotation of 358°) is likely the main factor contributing to this observation. For head and neck cancer patients with DMCs undergoing RT, VMAT would be a more robust beam delivery technique to reduce the DMC effect. This observation is congruent with previous cases evaluating the impact of dental implants.^(13)^

For single beam irradiation, Farahani et al.^(22)^ reported that the dose enhancements on the backscatter side adjacent to the various dental metals were 70% for gold, 60% for Ag‐Hg amalgam, and 40% for Ni‐Cr. Spirydovich et al.^(10)^ also reported an approximately 50% scatter dose increase at the incident surface from dental amalgam (mass density: $8.76\;g\;{\text{cm}}^{-3}$) and high‐gold alloy (mass density: $11.7\;g\;{\text{cm}}^{-3}$). In a recent study, Shimamoto et al.^(15)^ reported increases in the scatter dose from silver‐palladium‐gold alloy (50% Ag, 20% Pd, 14.4% Cu, 12% Au, 3.6% Ir+Zn+In), Ag alloy (72% Ag, 13% Zn, 9% Sn, 6% In), cobalt‐chromium alloy (52% Co, 25% Cr, 14% W, 8% Ga, 1% Al), and nickel‐chromium alloy (78.8% Ni, 19.5% Cr, 1.1% Si, 0.4% Fe, 0.2% Al) of 16.6%, 15.8%, 8.5%, and 7.3%, respectively, at the incident surface of the materials. For IMRT and VMAT techniques in this study, the dose differences between the measured and calculated doses to the area adjacent to the DMC were −9.2%–32.1% for IMRT and 3.1% –19.8% for VMAT. Shimamoto and colleagues^(15)^ reported that the scatter doses from various dental metals increased by only 1.4%–4.3% for IMRT, and Mail et al.^(16)^ reported a 9.25% increase in the backscatter dose from a dental filling material with VMAT. Generally, the scattered doses from dental alloys depend on the effective atomic number.^7^, ^15^ In addition, measurement parameters (i.e., photon energy, field size, beam incident angle, size and shape of the material, and atomic number of the material) affect the dosimetric impact of metal dental restoration materials.

Several studies have evaluated techniques to correct the dose disturbances caused by metal dental restoration materials. One such proposed method was the use of metal artifact‐corrected imaging data.^13^, ^23^, ^24^ Alternatively, Mail et al.^(16)^ suggested correcting target underdosing by using a virtual filter around the dental filling materials in the TPS calculation. We evaluated whether a relationship could be determined to enable an estimation of the dose disturbance caused by DMC. However, there were no strong correlations between the calculated doses and the measured dose enhancements. We concluded that it was difficult to estimate the magnitude of dose perturbations based on the TPS calculated doses using the traditional dose calculation algorithms such as AAA. In a recent study on a metallic hip implant, a newly introduced dose calculation algorithm (e.g., Acuros XB; Varian Medical Systems) was shown to have good agreement with measurements and full Monte Carlo simulations in the vicinity of the implant.^25^, ^26^ In future, we plan to evaluate these advanced dose calculation algorithms for metal dental restoration materials. In contrast, clear correlations between the average calculated dose on both sides of the DMC and the average dose enhancements on both sides of the DMC were observed. These results suggest that the effects of the interaction between dose enhancement and reduction were seen for both IMRT and VMAT plans. However, especially for IMRT, the frequency of these effects will depend on the incident beam angle, as mentioned above.

In the present study, the mock DMC had little impact on the measured dose at the downstream mid‐ and deep‐depth distance from the DMC. However, dose reduction is a well‐known phenomenon for a beam penetrating through the metal dental restoration materials. Spirydovich et al.^(10)^ showed that the predicted calculated dose downstream of high‐density dental restoration materials was higher than the measured dose by 10%–16%. For VMAT, Lin et al.^(14)^ showed that the presence of dental implants degraded the planned target volume coverage. Our results differ from those of the Spirydovich and the Lin studies likely because of the differences in the shape and size of the dental restoration materials. Specifically, our phantom imitated the full cast crown, including the inside teeth, whereas those used by Spirydovich et al. and Lin et al. imitated the dental filling material and the dental implant, both of which are completely filled with the material. Thus, dose reduction downstream from the high‐density dental restoration materials strongly depends on the characteristics of the dental restoration materials, and should be taken into consideration during the treatment planning process.

**Figure 10:**
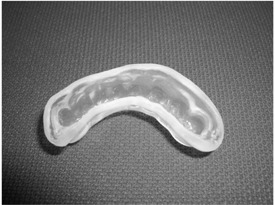
Sample picture of an individual intraoral mouthpiece.

At our institution, one method for resolving the dose enhancement caused by metal dental restoration materials is to employ an individual intraoral mouthpiece that maintains a space between the mucosa and metal materials. Figure 10 depicts an example of a mouthpiece created during preradiotherapy dental management. In the experimental oral phantom used in this study, we confirmed the capability of a mouthpiece to spare the oral mucosa from dose enhancement. However, the range of dose enhancement for various types of dental restoration materials is still debatable. Das and Kahn^(9)^ showed that the backscatter effect only persists for a few millimeters (i.e., dose enhancement reduced to 12% at 3 mm from 70% at 0 mm upstream from a lead block). Moreover, Das et al. and Spirydovich et al. referred to the fact that the dose enhancement just upstream from regions of high‐density dental materials was less susceptible to the thickness of the high‐density dental materials.^9^, ^10^ In addition, while only few studies have ever evaluated sparing the oral mucosa from dose enhancement, Mail and colleagues^(16)^ demonstrated the clinical usefulness of a 3 mm custom‐made plastic dental mold. Based on the results of this study and previous studies, a mouthpiece thickness of 3 mm seems clinically appropriate for various types of dental restoration materials.

A limitation of this study is that the physical density of the mock teeth embedded in the experimental oral phantom is slight different from that in other studies. The physical density of the teeth for implementing the Monte Carlo simulation was $2.2\;g\;{\text{mc}}^{-3}$ in the study by De Conto et al.^(11)^ and $2.2\;g\;{\text{cm}}^{3}$ in the study by Chang et al.^(27)^ In fact, the CT number of the mock teeth was lower than that of human teeth (i.e., mock teeth: 619 HU, human teeth [Patient 1]: 1402 HU). While this limitation cannot be ignored, it may have had little impact on the results, as Mail et al.^(16)^ reported that the backscatter dose from teeth is insignificant compared with that from dental restoration material. Moreover, the CT number of the mock teeth enclosed by the DMC was corrected according to the CT number of the surrounding polymethyl methacrylate in the planning phase.

## V. CONCLUSIONS

Dose enhancements caused by DMC‐mediated radiation backscatter occurred during IMRT and VMAT. Because it is difficult to accurately estimate the dose perturbations, careful consideration is necessary when planning IMRT or VMAT for the treatment of head and neck cancer. In addition, to spare the oral mucosa from dose enhancement, the use of an individual intraoral mouthpiece should be considered.

## ACKNOWLEDGMENTS

This work was partially presented at the 56th Annual Meeting of the American Society for Therapeutic Radiology and Oncology (ASTRO) in San Francisco, September 14–16, 2014. This research was supported by a Grant‐in‐Aid for Young Scientists (B) from the Ministry of Education, Culture, Sports, Science, and Technology, Japan (Grant No. 15K21060).
